# Gradient acoustic focusing of sub-micron particles for separation of bacteria from blood lysate

**DOI:** 10.1038/s41598-020-60338-2

**Published:** 2020-02-28

**Authors:** David Van Assche, Elisabeth Reithuber, Wei Qiu, Thomas Laurell, Birgitta Henriques-Normark, Peter Mellroth, Pelle Ohlsson, Per Augustsson

**Affiliations:** 1 Department of Biomedical Engineering, Lund University, Ole Römers väg 3, 22363 Lund, Sweden; 2 CNRS, Univ. Bordeaux, CRPP, UMR 5031, 115 Avenue Schweitzer, 33600 Pessac, France; 3 Department of Microbiology, Tumor and Cell Biology, Karolinska Institutet, 171 77 Stockholm, Sweden; 4 Department of Clinical Microbiology, Karolinska University Hospital, 17176 Stockholm, Sweden; 5 Singapore Centre for Environmental Life Sciences Engineering SCELSE and Le Kong Chian School of Medicine LKC, Nanyang Technological University, NTU, 50 Nanyang Ave, 639798 Nanyang, Singapore

**Keywords:** Fluid dynamics, Biomedical engineering, Bacterial infection

## Abstract

Handling of submicron-sized objects is important in many biochemical and biomedical applications, but few methods today can precisely manipulate this range of particles. We present gradient acoustic focusing that enables flow-through particle separation of submicron particles and cells and we apply it for separation of bacteria from blood lysate to facilitate their detection in whole blood for improved diagnostics. To control suspended objects below the classical 2µm size limit for acoustic focusing, we introduce a co-flowing acoustic impedance gradient to generate a stabilizing acoustic volume force that supresses acoustic streaming. The method is validated theoretically and experimentally using polystyrene particles, *Staphylococcus aureus*, *Streptococcus pneumoniae* and *Escherichia coli*. The applicability of the method is demonstrated by the separation of bacteria from selectively chemically lysed blood. Combined with downstream operations, this new approach opens up for novel methods for sepsis diagnostics.

## Introduction

Microfluidic flow-through channel networks can be employed to separate or analyze biological particles by their fluorescent^1^, optical^2^, electrical^3–5^, magnetic^6,7^ or bio-mechanical properties^8–12^. A major benefit with these technologies is the ability to integrate sample preparation with optical or chemical analyses on the same chip. Today, few microfluidic tools can handle suspensions containing objects below 1 µm. The main reason is that small objects have a large surface-to-volume-ratio, which makes it challenging to generate a sufficient force to drag them swiftly through a liquid.

Acoustic fields can be employed inside microfluidic channels to generate strong acoustic radiation forces to spatially position suspended biological particles^13^. In acoustophoresis^14^ cells are separated by their lateral displacement in response to an acoustic field while flowing through a microfluidic channel. For particles smaller than 2 µm the particle motion is normally dominated by acoustic streaming^15–17^ which has been a major limitation for this technology. Several systems have been proposed that address this limitation using acoustic seed trapping^18^, acoustic vortices^19,20^ or a different aspect ratio of the acoustic cavity^21–24^. Purification of small particles, e.g. bacteria and platelets^25–28^ has been achieved by acoustically pushing away the larger particles from the smaller particles, but this approach does not enable isolation of sub-micron-sized biological particles from a complex background of molecules, particles or debris that are of smaller size, which is essential for purification protocols. Recently sub-micron particles were pushed away from 100-nm-particles by surface acoustic waves^29,30^ and the same approach was applied to separate red blood cells and microvesicles from exosomes in a two-stage process^31^.

We recently found that, for bulk acoustic waves, acoustic streaming can be greatly reduced by introducing a gradient in acoustic impedance (mass density times speed of sound) in the acoustic cavity by standard gradient centrifugation media^32,33^. Efficient suppression of streaming opens up for acoustic separation of particles below the classical size limit and thereby enables transfer of micrometer- and sub-micrometer-sized particles in continuous flow from one medium to another using acoustic radiation forces, inspiring new applications of bulk acoustic waves involving platelets, exosomes, large organelles and bacteria.

One such application is diagnosis of bacterial blood stream infections. The current gold standard method is based on culturing of a blood sample, which has an inherent drawback due to time-consuming incubation times of hours to days^34,35^. The timely initiation of a targeted therapy, however, is crucial for sepsis patients since survival decreases for every hour that an appropriate treatment is delayed^36^, emphasizing the demand for faster diagnostics. Several methods are available for direct nucleic acid extraction and detection using polymerase chain reaction (PCR), although none has yet been able to replace blood culture^37^. One reason for this is the low number of bacteria (frequently less than 200 colony forming units per milliliter blood) in human sepsis^38^. For faster blood stream infection diagnosis, viable bacteria can be purified and recovered by selective lysis and subsequent concentration by centrifugation^39,40^, commercially available as the Isostat^R^/Isolator^TM^ Microbial System, Abbott. Lysate centrifugation, as a blood culture independent method, leads to shorter time to identification in comparison to automated blood cultures^41,42^. However, the lysis-centrifugation method is not in routine clinical use, due to contamination risks and labor intensive handling^42^, which are hurdles that can be diminished by chip-integrated microfluidic approaches for blood cell lysis^43,44^ and sample concentration^26,45^.

Here we introduce **gradient acoustic focusing** (GAF) that enables separation of submicron particles via acoustic transport from one medium to another by suppressing the acoustic streaming using acoustic impedance gradients. We apply this method for acoustic focusing of three different bacterial species to perform efficient medium exchange and we demonstrate chip-integrated sample preparation by separating bacteria from lysed blood.

## Theory of Operation

**Figure 1:**
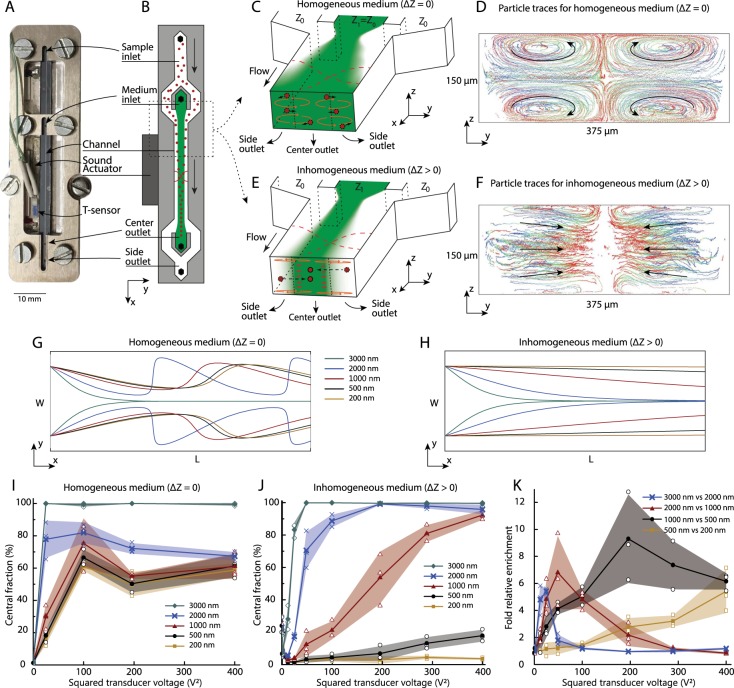
The working principle of gradient acoustophoresis. **(A)** A photograph of the acoustofluidic chip in its holder. **(B)** A schematic of the GAF separation principle. **(C)** Schematic illustrating mixing induced by acoustic streaming when the acoustic impedances of the central medium (Z_1_) and the particle suspension (Z_0_) are perfectly matched (i.e. ΔZ = (Z_1_ − Z_0_)/Z_1_ = 0). **(D)** Positions of 1-µm-diameter particles, measured by 3D tracking during stop flow for 20 s, projected onto the cross sectional *y*-*z*-plane, for ΔZ = 0, illustrating mixing by acoustic streaming. The elapsed time after onset of sound is indicated by colors ranging from blue (t = 0 s), via green (t = 10 s), to red (t =20 s). **(E)** Schematic illustrating the suppression of acoustic streaming that enables particle translocation to the central flow by forming an acoustic impedance gradient (ΔZ > 0) when injecting aqueous solution of Ficoll through the central inlet branch. **(F)** Positions of 1-µm-diameter particles, measured by 3D tracking during stop flow for 20 s, projected onto the cross sectional *y*-*z*-plane, for ΔZ > 0, illustrating suppression of acoustic streaming. The elapsed time after onset of sound is indicated by colors ranging from blue (t = 0 s), via green (t = 10 s), to red (t = 20). **(G)** Simulated trajectories of particles that are subjected to both acoustic radiation forces and acoustic streaming, for an acoustic energy density of 250 J m^−3^ and **(H)** particles that are separated in a streaming-free environment. **(I)** The central fraction of recovered particles (divided by all recovered particles) after separation vs ultrasound actuator voltage amplitude, for homogeneous medium (ΔZ = 0). **(J)** The central fraction of recovered particles (divided by all recovered particles) after separation vs ultrasound actuator voltage amplitude, for inhomogeneous medium (ΔZ > 0), N = 3. **(K)** Fold relative enrichment of particles relative to a smaller particle size vs ultrasound actuator voltage amplitude for inhomogeneous medium (ΔZ > 0). Same dataset as in **(J)**. **(I–K)** Open symbols indicate data points, filled symbols indicate average and shaded fields indicate standard deviation (N = 3).

In free flow acoustophoresis (Fig. 1A,B) suspended objects are separated based on their differences in acoustic mobility due to their size^14,46–51^ and acoustic contrast^8,52–54^ relative to the suspending medium^55–57^. The suspension is continuously processed through a microfluidic separation channel where an acoustic standing wave or a leaky surface acoustic wave deflects the objects transversely to the flow. Cell-free medium is injected in the central branch of a trifurcation inlet to position the objects near the walls upon entering the channel. At the end of the channel, the flow is split in a trifurcation outlet so that objects of high acoustophoretic mobility exit through the central outlet and objects of low, zero or negative mobility exit to the side channels.

The lateral deflection of objects in a half standing wave acoustophoresis channel is caused by acoustic radiation forces (Supplementary Fig. 1A) which originate from sound scattering on the suspended objects. The acoustic-radiation-induced velocity *u*_rad_ (Eq. 1) exerted on a spherical object by the acoustic field can be expressed

1 $${u}_{{\rm{r}}{\rm{a}}{\rm{d}}}\propto {E}_{{\rm{a}}{\rm{c}}}\Phi (\mathop{\rho }\limits^{ \sim },\mathop{\kappa }\limits^{ \sim }){a}^{2}/\eta $$

where

2 $${E}_{{\rm{ac}}}=\alpha {U}^{2}$$

for (*E*_ac_) the resonator’s acoustic energy density, (*a*) the object’s radius, (Φ) the contrast factor that depends on the relative density ($\tilde{\rho }$) and compressibility ($\tilde{\kappa }$) of the object with respect to the suspending medium, and (η) the dynamic viscosity of the suspending medium. The acoustic energy density can be tuned by altering the electrical signal amplitude (*U*) applied to the piezoceramic actuator that vibrates the chip with (α) being a system dependent factor (Eq. 2).

A second effect that influences the trajectories of objects in acoustophoresis is acoustic streaming that originates from viscous dissipation of energy inside the thin (<1 µm) boundary layers at the channel floor and ceiling when oscillatory sound waves propagate along these surfaces. This causes steady conveyor belt-like flows directed towards the channel sidewalls that generates a recirculating flow in the bulk^15^ (Supplementary Fig. 1B). Such streaming has been extensively studied numerically^21^ and experimentally^16,17^, for the current channel geometry.

The interplay between radiation forces and acoustic streaming determines the overall lateral velocity of particles in acoustophoresis. The radiation-induced velocity *u*_rad_ is dominant for larger particles, while the streaming-induced velocity *u*_str_ dominates for smaller particles. The critical particle diameter (2*a*_c_), i.e. the crossover point from radiation-dominated to streaming-dominated particle motion, can be determined by equating u_rad_ and u_str_^16^.

3 $$2{a}_{c}=\,\sqrt{\frac{12\,s\,\eta }{\pi f\varPhi \rho }}\approx 1.4\,\mu m$$

for η/ρ = 0.89 mm²/s, Φ = 0.17, sound frequency (*f*) = 2 MHz and a geometry dependent factor (s) = 0.194. Values are given for a polystyrene particle suspended in water.

Different approaches have been proposed to reduce the effect of acoustic streaming and in this way reduce the critical radius. Equation 3 shows that the critical radius can be lowered by operating at a higher frequency, changing the properties of the medium (η, ρ, κ) or adjusting the channel geometries (s)^16,21–24^.

Stabilizing acoustic body forces in the bulk^58^ can suppress acoustic streaming when miscible fluids of different acoustic impedance (Z) are laminated side by side in an acoustophoresis channel. For such inhomogeneous systems, the liquid of highest acoustic impedance strives to relocate towards the acoustic pressure nodes^59,60^. We recently established that laminated fluids that forms an acoustic impedance gradient efficiently counteract the recirculating flow in the bulk and thereby confines the streaming rolls to the channel floor and ceiling. This enables streaming-free particle manipulation based on acoustic radiation below the critical size^32,33^. Building on this, we herein investigate GAF, which is an approach to separate objects below the critical size through stabilizing volume forces in the bulk. This is achieved by tailoring the acoustic properties of the suspending medium by creating a gradient in acoustic impedance across the width of the separation channel. An important feature of GAF is that the gradient in acoustic properties must be sufficiently weak so that the particles maintain the same sign of their acoustic contrast when moving through the gradient. This contrasts to another gradient-based method, iso-acoustic focusing, where cells are being focused to their iso-acoustic point, where they have zero acoustic contrast, in an acoustic impedance gradient^8^.

## Results

### Working principle

In GAF acoustic streaming is greatly suppressed which enables separation of sub-micron particles in a flow-through process. Figure 1A shows a photograph of the GAF unit with the etched silicon microfluidic structure and a piezoelectric actuator glued to the back. Particle suspension enters through the side branches of a trifurcation structure and particle free medium is injected through the central branch, Fig. 1B. Flow rates are given in Supplementary Table 1. The acoustic impedance of the central liquid (Z_1_) can be increased relative to the particle suspension (Z_0_) by adjusting the concentration of an aqueous solution of hydrophilic polysaccharide (Ficoll). When the liquids laminate at the junction, an acoustic impedance gradient forms across the acoustic separation channel that gradually flattens out by molecular diffusion during the passage through the device. Particles are transported from the regions near the walls towards the central flow by acoustic radiation forces exerted by a half wavelength ultrasound standing wave directed orthogonal to the flow in the plane of the chip.

For a homogeneous system of liquids with matched acoustic impedances (ΔZ = (Z_1_ − Z_0_)/Z_1_ = 0), the sound induces a steady acoustic streaming flow in the channel which leads to mixing of the incoming flow streams, Fig. 1C. To confirm that acoustic streaming exists in the bulk of the separation channel for the homogeneous system, we performed particle tracking in 3D of 1-µm-diameter particles using General Defocusing Particle Tracking^61^ for a 1 mm segment of the channel after temporarily stopping the flow. The temporal evolution of the particle positions, projected onto the cross sectional (*y*-*z*) plane, reveals four acoustic streaming rolls that efficiently mix the particles and prevent focusing of particles by acoustic radiation forces, Fig. 1D and Supplementary Movie 1.

For an inhomogeneous system of liquids that form a gradient in acoustic impedance, such that ΔZ > 0, an acoustic volume force is generated in the bulk of the liquid, which stabilizes the position of the central high impedance liquid^58^. This stabilizing volume force suppresses the acoustic streaming rolls in the channel by holding the liquid in place and confining the streaming rolls to the floor and ceiling of the channel^32^, Fig. 1E. This drastically reduces the acoustic streaming driven mixing and enables sub-micron particle focusing by acoustic radiation forces. Particle tracking in stop-flow reveals efficient confinement of the streaming rolls to the channel floor and ceiling that enables focusing of 1-µm-diameter particles near the channel center, Fig. 1F and Supplementary Movie 2.

Simulated particle trajectories (Supplementary Note 1) for the homogeneous system show that particles below 2 µm in diameter follow spiraling trajectories, Fig. 1G, that prevents fractionation of particles into distinct populations. When setting the acoustic streaming component to zero, the particles focus to the center at a size dependent rate, Fig. 1H.

We investigated the effect of increasing the acoustic impedance of the central inlet branch medium when focusing sub-micron polystyrene particles in the acoustic field. Water or an aqueous solution of Ficoll, was introduced through the central inlet branch and samples were processed at different acoustic energy densities. Both outlets fractions were analyzed by flow cytometry to compare the relative fraction of particles of each size that were collected through the central outlet compared to all collected particles of each size, Fig. 1I,J.

For the homogeneous system (Fig. 1I) having water as central medium, particles below 2000 nm in diameter did not separate from each other and would not focus completely in the central outlet. Due to the acoustic streaming rolls in the bulk, these streaming-dominated particles all have a similar central fraction for increasing acoustic energy since they primarily follow the streaming rolls that moves the particles initially to the center and then out to the sides again, Fig. 1G. The 3000-nm-particles, being larger than the classical theoretical critical diameter for the crossover between streaming and radiation dominated particle motion, translocate to the central outlet already for the lowest level of acoustic actuation. For the 2000-nm-particles, which are near the critical diameter, the contribution from radiation and streaming is of the same magnitude, which offsets them from both the radiation-dominated and the streaming-dominated bead sizes.

For the inhomogeneous medium, having Ficoll (5% w/v) as central medium, a clear size-dependent separation was observed where an increase in applied voltage caused a monotonically increasing recovery of beads in the central outlet for all bead sizes (Fig. 1J). For high amplitudes, the 200-nm-particles remain in the side outlet while 1000-nm-particles are completely focused to the central outlet branch, indicating that the acoustic streaming is suppressed and that the acoustic field is sufficiently strong to enable transport of bacteria-sized objects across flow streams within seconds. The trend for the 500-nm-particles shows that these particles are partially focused and could be separated from 200-nm-beads by further increasing the acoustic field amplitude. We calculated the enrichment factors for each particle size by comparing the central outlet concentration with the inlet concentration. By taking the ratio of the enrichment factors for different combinations of particles, we derived the fold relative enrichment for the particles compared to particles of the adjacent smaller size. Figure 1K shows that each particle size can be enriched 5- to 10-fold compared to its neighboring smaller particle size by proper tuning of the acoustic field amplitude. This implies that a subset of a particle mixture can be extracted by size discrimination in a two-step procedure.

For zero acoustic energy, in the inhomogeneous configuration, a small fraction of the particles is detected in the center due to a gravitational effect, Fig. 1J. A slight mismatch in density between central and side liquid rearranges the less dense suspension, containing the particles, to the top of the channel. Already for the lowest level of applied acoustic actuation, this gravity effect is countered by acoustic volume forces in the bulk, which has been previously described^8,58,59^.

The overall recovery of particles retrieved through both outlets relative to the input sample was, for homogeneous media, 79.6 ± 10.8%, and for the inhomogeneous media, 77.2 ± 10.0% (Supplementary Table 2). The recovery is adjusted to account for the particles remaining in the system after stopping the flow when the input test tube is emptied. We computed this anticipated loss to be 18% by estimating the internal swept volume of the sample’s path from the inlet to the outlet relative to the processed sample volume. The relative loss of particles can be decreased by minimizing the internal volume or by increasing the sample volume.

### Tailoring the acoustic impedance gradient

It is critical in GAF to properly configure the acoustic impedance gradient across the separation channel. The difference in acoustic impedance between the side and center inlet must be large enough to counter acoustic streaming by acoustic volume forces. However, adding in higher concentrations of Ficoll, or other substances that alter the acoustic impedance, typically decreases the acoustic mobility of suspended objects due to an increase in viscosity of the medium and lower acoustic contrast.

**Figure 2:**
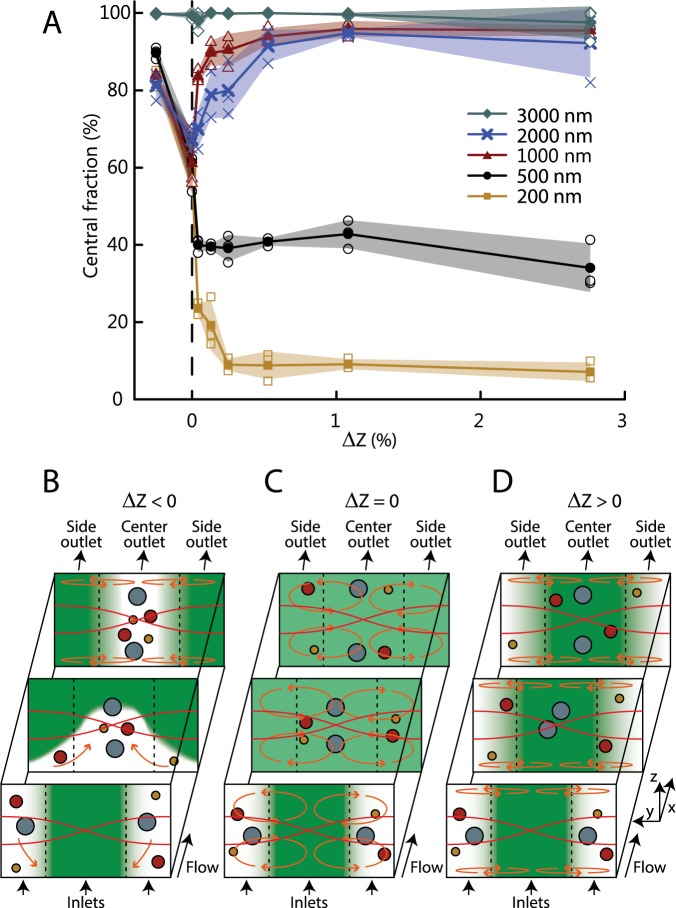
Tailoring the acoustic impedance gradient. **(A)** Plot of collected central fraction against ΔZ (open symbols). Filled symbols indicate the averages and shaded fields indicate the standard deviations (N = 3). **(B)** Schematic of acoustic relocation of fluids (orange arrows) in the acoustic field (red lines) of the particle suspension (filled circles) when ΔZ < 0, i.e. for Z_center_ < Z_side_, causing particles to gather in the center of the channel. **(C)** Schematic of acoustic streaming causing mixing in the case of a homogeneous medium, i.e. for ΔZ = 0. **(D)** Schematic of the acoustic streaming suppressed by bulk acoustic forces when ΔZ > 0 enabling size-based separation.

We investigated the effect of the impedance gradient magnitude by varying the Ficoll concentration of the central inlet particle-free medium, Fig. 2A. For negative relative impedance difference (ΔZ = (Z_center_ − Z_side_)/Z_side_ < 0) all particle sizes are recovered through the central outlet, but are not separated, and this is due to relocation of the suspending medium itself by acoustic volume forces, Fig. 2B^58–60^. For matched impedances (ΔZ = 0) the central fraction drops and the particles do not separate due to mixing by acoustic streaming, Fig. 2C. All positive impedance differences improved separation of particles for the investigated range (ΔZ = 0.1% to 2.7%) due to the stabilizing acoustic volume force, Fig. 2D. For ΔZ > 1% the separation reaches a stable configuration in the investigated range with no statistical change (t-statistics, α = 0.05). The overall recovery for all particles sizes was 75.1 ± 10.8% (Supplementary Table 3).

### Optimizing the flow

In GAF the particles describe 3-dimensional trajectories that depend on the microchannel flow velocity profile and the acoustic streaming and radiation in the plane transverse to the flow. In addition, during the passage of the channel, the acoustic impedance gradient flattens by molecular diffusion affecting the particle transport. Therefore, a given media configuration can have an optimal combination of acoustic field amplitude and flow velocity that maximizes the transfer of a particle of a certain size, which is not necessarily obtained by low flow and high acoustic field. To test this, the characteristic passage time (*t*_pass_) through the channel was varied by adjusting the flow rate (*Q*) such that *t*_pass_ = *H* ⋅ *W* ⋅ *L*/*Q*, where *H*, *W* and *L* is the channel height, width and length respectively. The acoustic driving amplitude (*U*^2^ = 400 V^2^) and acoustic impedance gradient (Δ*Z* = 2.76%) were kept constant.

**Figure 3:**
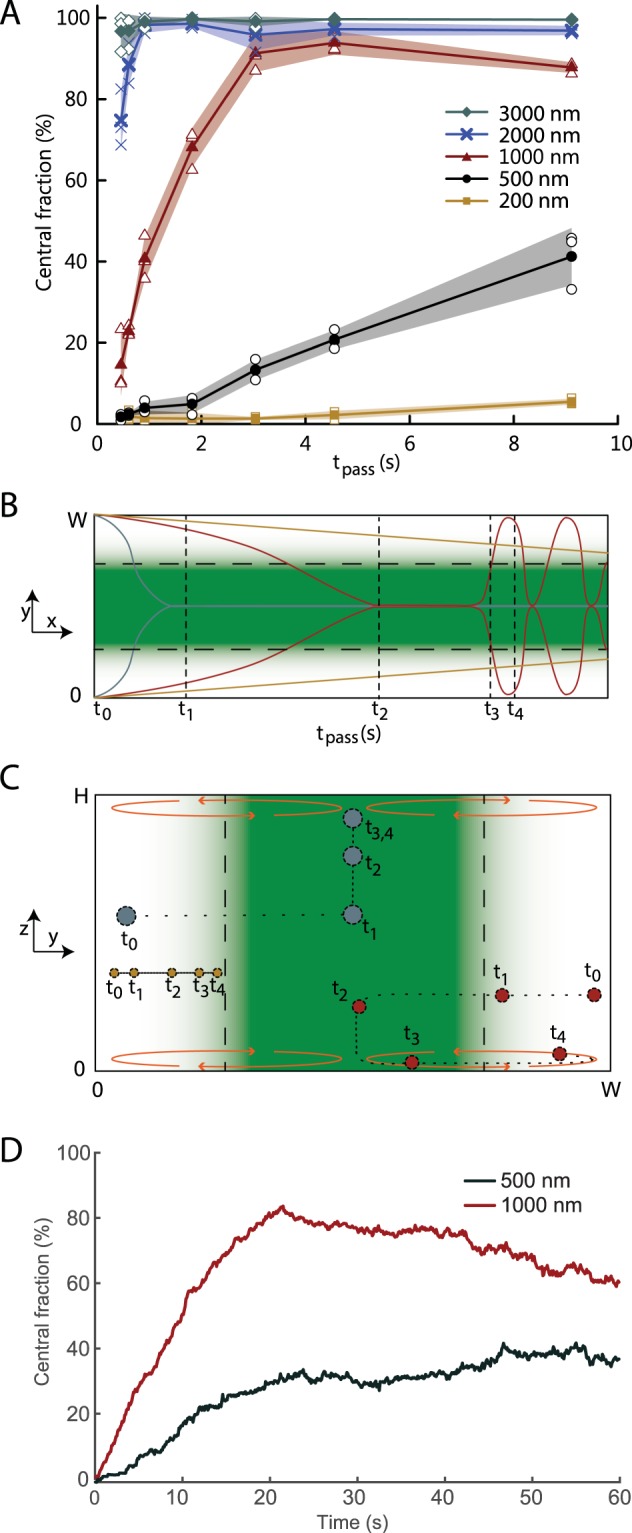
Optimizing flow. (**A**) Plot of collected central fraction against the average passage time (t_pass_) through the channel (open symbols). Filled symbols indicate the averages and shaded fields indicate the standard deviations (N = 3). **(B,C)** Schematics of the trajectories of a large (blue), intermediate (red) and small (orange) particle at different times after entering the separation channel as viewed **(B)** from the top and **(C)** along the flow. **(D)** Plot of central fraction vs time for 500-nm-particles and 1000-nm-particles by analyzing the stop flow data for particle positions shown in Fig. 1F, for ΔZ = 2.45%. The time scales in **(A)** and **(D)** are different because 3D tracking could not be carried out at high acoustic energy densities.

Figure 3A shows that the 1000-nm-particles, for short *t*_pass_ do not have sufficient time to congregate in the channel center, and reach the most favorable transfer to the central flow stream for *t*_pass_ ≈ 5 s. The observed drop for longer *t*_pass_ is likely caused by particles becoming trapped in the flat flow rolls near the channel floor and ceiling after first having reached the channel center, Figs. 1F and 3B,C. Eventually, after 30–60 s, outside the investigated range, the flow streams become homogenized due to molecular diffusion (D_Ficoll_ = 2,7.10^−11^ m²/s, Supplementary Note 2) and mixing by acoustic streaming^32^. We further analyzed the stop-flow particle tracking data from Fig. 1F by computing the relative number of particles in the central region (width **0.4** ***W***) and plotted the development of the central fraction of particles over time for the 500-nm- and 1000-nm-particles (Fig. 3D, Supplementary Fig. 2 and Supplementary Movies 1 and 2**)**. Similar to the flow data, the 1000-nm-particles reach a peak and then drop off while the 500-nm-particles do not reach a maximum in the investigated range.

### GAF of bacteria

Due to their small size, bacteria are challenging to focus by standard free flow acoustophoresis^19^. To test if GAF is a viable route to achieve focusing and separation of bacteria we studied acoustic focusing of three bacterial species; *Staphylococcus aureus*, *Streptococcus pneumoniae* and *Escherichia coli*, as relevant pathogens from a sepsis diagnosis perspective^36^.

First, we confirmed that bacteria could not be selectively collected in the center outlet when the above-described set-up is operated with standard homogenous medium of invariant acoustic impedance. Only 59.9 ± 12.0% (N = 3) of *S. aureus* and 63.7 ± 9.4 (N = 3) of the spiked-in 1000-nm-particles were collected through the central outlet branch, corresponding well to the previous observation for 1000-nm-beads (Fig. 1I).

**Figure 4:**
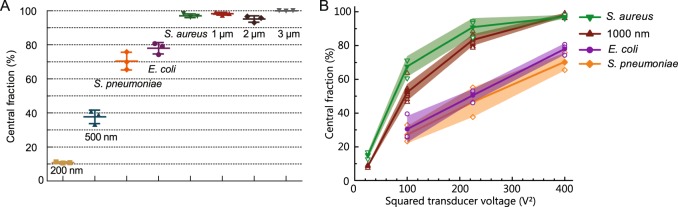
GAF of bacteria. **(A)** *S. aureus, S. pneumoniae* and *E. coli* suspended in water with 2 g/L BSA were focused with Ficoll (5% w/v) as central medium at a fixed actuation amplitude (400 V^2^). Horizontal lines indicate average and standard deviation (N = 3). Focusing of polystyrene beads is shown for reference. **(B)** Focusing of bacteria for a range of actuation voltages (open symbols). Filled symbols indicate averages and shaded fields indicate standard deviations (N = 3).

By applying GAF to bacterial solutions, however, it was possible to achieve focusing of the gram-positive *S. aureus* and *S. pneumoniae* as well as of the gram-negative *E. coli*. Thus, in the inhomogenous media of the above described GAF set-up with a 5% Ficoll solution in the central inlet (ΔZ = 2.7%) *S. aureus* could now be collected at 97.0 ± 0.9% in the center fraction and *E. coli* and *S. pneumoniae* at 80.0 ± 2.7% and 70.4 ± 4.2%, respectively (Fig. 4A). Compared to the separation of beads in GAF, the spherically shaped *S. aureus* showed focusing characteristics similar to 1000-nm-beads, whereas the rod-shaped *E.coli* and coccoid *S. pneumoniae* focused to a lower degree as compared to 1000-nm-beads, though with a higher ratio in the center outlet than 500-nm-beads. These differences in response to the applied acoustic actuation of the different bacterial species remained consistent for all investigated sound amplitudes (Fig. 4B). Percentage of recovered bacteria and beads are given in Supplementary Tables 4 and 5.

The reason for the different behavior of the bacterial species in response to the acoustic standing wave could neither be attributed to differences in the mean volume of the respective bacterial population as measured by electrical resistance volumetric sizing (Supplementary Fig. 3), nor to their densities as measured by sink-float analysis in density gradient medium (Supplementary Fig. 4 and Supplementary Table 6). Besides the to-date unknown bacterial species-specific differences in compressibility, the characteristic bacterial response to acoustic actuation could reflect variations in viscous drag attributable to the distinct bacterial morphology. The differences in size distribution of the investigated bacterial populations could also reflect the tendency of inter-bacterial adhesion and pneumococcal chain formation (Supplementary Fig. 3).

### Bacterial isolation from blood through selective lysis followed by GAF

As shown above, GAF provides the possibility to influence the migration of a collection of sepsis-causing bacteria and achieve efficient separation from objects smaller than 200 nm (Fig. 4**)**. These findings indicate that GAF could potentially be used to perform in-line particle separation and medium exchange in diagnostic and analytical instrumentation. As an example, we suggest a novel method for bacteremic blood sample preparation through selective lysis of the blood cells followed by separation of the bacteria from the lysate using GAF. In combination with downstream bacterial detection, identification and antibiotic resistance profiling, this could become a faster alternative for bacterial blood stream infection diagnosis.

**Figure 5:**
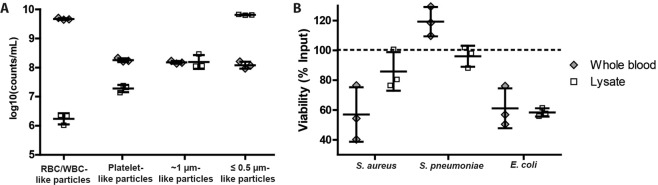
Lysate characteristics. **(A)** Analysis of cell and particle distribution in whole blood versus blood lysate. Cells, platelets and cell debris of different sizes were analyzed using flow cytometry, with a protocol visualized in Supplementary Fig. 5. Decimal logarithm of particle counts per mL sample in the forward scatter (FSC) gates that characterize the respective blood cells (RBC = red blood cells, WBC = white blood cells, platelets) in whole blood. Furthermore, particles that had FSC characteristics between the ones equivalent to 0.9-µm-doublets and 0.5-µm-particles of the reference beads (Megamix plus FSC kit, BioCytex) were grouped together as ~1 µm-like particles, whereas ≤0.5 µm like particles comprised those with a FSC equivalent to 0.5-µm-particles and below as long as they were clearly distinguishable from the noise. **(B)** Viability in percent of the respective bacteria in blood lysate and control (PBS) treated whole blood compared to bacteria incubated in nutrient broth (=100%). Values for averages and standard deviations are indicated by horizontal lines (N = 3).

First, a suitable selective lysis agent was selected. Combinations of agents that have been described to cause blood cell lysis were examined for their efficiency to selectively lyse blood cells without adversely affecting bacterial viability to allow subsequent growth dependent antibiotic susceptibility testing (Supplementary Note 3, Supplementary Table 7). As for most of the tested combinations, the lysis protocol selected for the following GAF experiments reduced the number of large blood cells and platelets by several orders of magnitude and did not result in high particle numbers of about 1 µm in size overlapping with the bacterial size range, Fig. 5A. Importantly, the selected blood lysis procedure was not adversely affecting viability of *S. pneumoniae*, *S. aureus* and *E. coli* that were spiked into the blood, Fig. 5B.

With the above observed relationship between decrease of focusing efficiency with decreasing particle size, Fig. 4A, GAF could constitute an efficient procedure to separate the bacteria from the high number of <0.5 µm particles that are produced during lysis (Fig. 5A).

To avoid relocation of the whole sample liquid to the center and to suppress acoustic streaming during GAF, a central medium was selected with an acoustic impedance ~1% above that of the sample stream (Supplementary Table 8) as this was shown above to be a prerequisite for effective particle focusing in stratified media (Fig. 2A).

**Figure 6:**
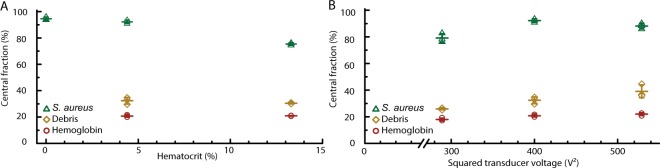
Separation of bacteria from blood lysate. **(A)** Focusing efficiencies of *S. aureus* spiked into water with 0.2% BSA (0% hematocrit), 3× diluted lysate (4.4% corresponding hematocrit) or undiluted lysate (13.3% corresponding hematocrit) using an actuator voltage of 400 V^2^. Fraction of *S. aureus* (green), debris (yellow) and hemoglobin (red) collected in the center channel in percent of the total amounts collected after passage through the microfluidic device. **(B)** The influence of varied applied actuation amplitude on focusing efficiencies for 3× diluted lysate samples. Horizontal lines indicate values for averages and standard deviations of triplicate measurements.

Our data showed that *S. aureus* was focused to a similar extent from water with 2 g/L BSA into the central media stream with 2% and 5% Ficoll (Figs. 4A and 6A). Indeed, even for more viscous samples, such as three-fold diluted blood lysates, we found that the focusing of *S. aureus* was performed with comparable efficacy (Fig. 6A). When processing undiluted lysate, the focusing efficiency decreased by 17 percentage points for *S. aureus* (Fig. 6A). This decreased focusing efficiency was likely attributed to the increased viscosity and reduced acoustic contrast factor in both the sample and center medium, lowering the particle velocity.

Reduction of debris from lysates was achieved by about 70% for both lysate concentrations. Only about 20% of the hemoglobin were transferred to the central outlet (Fig. 6A) indicating a five-fold dilution of the lysis buffer and antibacterial factors of the blood sample such as host immune defenses or antibiotics of an already initiated therapy^39^ when assuming similar diffusion coefficients, without increasing the sample volume. The lysate debris could be further reduced by lowering the separation voltage at the cost of decreased bacteria focusing (Fig. 6B). Higher separation voltage did not increase the focusing of bacteria, but generated higher debris contamination in the center fraction. Percentage of recovered bacteria and beads are given in Supplementary Tables 9 and 10.

## Discussion

We have presented and provided experimental and theoretical validations of the gradient acoustic focusing (GAF) method to perform size-based separation of a range of submicron particles by acoustic translocation from one medium to another. Our results show that sub-micron particle separation can indeed be carried out by in-line processing at a throughput of 12 µL/min, and we observed a 5-fold relative enrichment of 500-nm-particles relative to 200-nm-particles, Fig. 1K. The trend for the 500-nm-particles in Fig. 3A suggests that a further increase of the acoustic amplitude would enable complete focusing of particles below 500 nm. An increase in acoustic amplitude by two orders of magnitude will place this technology alongside nano DLD^62^ that has been demonstrated for size based displacement of exosomes. Compared to nano DLD, GAF is non-contact and less prone to clogging and does not require high pressures to operate. To increase the resolution of particle separation, particles could be pre-aligned by hydrodynamic focusing or by employing acoustic pre-alignment by a 2D-acoustic field^19,20^ prior to entering the GAF-separator. Besides the promising application of the size selectivity of GAF shown here for bacteria separation from blood lysates, a successful implementation of GAF in the 100 to 500 nm range would facilitate enrichment, medium exchange and size fractionation for exosomes, viruses and organelles. This would make GAF an automatable flow-through alternative to ultra-centrifugation and affinity columns, additionally facilitating handling of small sample volumes.

Many label-free microfluidic methods for bacteremic blood sample preparation rely on direct size-based separation without prior lysis^26,27,45,63–69^. Besides the reduction of blood cells of larger or similar diameter than the targeted bacteria and the potential release of phagocytosed but viable bacterial cells^38^, other benefits might arise from selective blood cell lysis as a first step in sample preparation. Thus, beside their interaction with leucocytes, bacteria can bind to platelets directly through surface receptors or indirectly via plasma protein linkage^70,71^. Moreover, complement-decorated bacteria have been described to attach to erythrocytes via the immune adherence mechanism^72–74^. By selectively lysing the eukaryotic cells these adhesive complexes are presumably disrupted or decreased in size, facilitating a discriminative size based separation of bacteria.

The here presented combination of selective blood cell lysis with GAF enables label-free separation of bacteria from smaller lysis debris, constituting a sample preparation method with potential to facilitate downstream bacteria detection, identification and resistance profiling. To achieve further debris removal or bacteria concentration, multiple GAF steps may be introduced. To increase throughput, GAF channels may be parallelized, extended in length, or the acoustic field amplitude can be increased. Furthermore, our suggested microfluidic approach can be adapted for operating in an automated, closed, flow-through analysis system reducing manual handling and associated contamination risks.

## Materials and Methods

### Device fabrication and instrument set-up

The acoustophoresis chip used in this study has been described previously^49^. In brief, a 150 µm deep channel structure was etched in silicon (wafer thickness ~350 µm) and sealed by anodic bonding to a glass lid (thickness ~1.1 mm). The chip dimensions are 3 mm by 90 mm and the separation channel is 27 mm long and 375 µm wide. The piezoceramic actuator, resonant at 2 MHz, was glued to the chip underneath the separation channel. To monitor the temperature, a PT-1000 resistance temperature detector was glued onto the actuator on its protruding side (Fig. 1A). Cooling was achieved by directing a stream of pressurized air onto the chip. The actuator was actuated using a function generator (AFG3022B, Tektronix) and an in-house developed power amplifier based on a LT1012 chip (Linear Technology). The applied signal was monitored using an oscilloscope (TDS2002C, Tektronix).The operating frequency was 1.89 MHz.

Two different flow set-ups were used in the experiments. The pressure-driven flow set-up uses an 8-port 0–2 bar proportional pressure control valve terminal (VEMA, FESTO) in combination with three liquid flow sensors (SLI-1000, Sensirion) to control the flow in a closed loop. The channel inlets and outlets were air-sealed and fluids were pressurized and injected through the inlets. The readout of the three sensors (at the center inlet and at the outlets) was processed using in-house developed flow control software written in LabView, which used a closed feedback loop. The flow set-up used to collect data in Fig. 3A, has three syringe pumps (neMESYS, CETONI GmbH). The three syringes were connected to the outlets and to the central inlet. The side inlet pulls sample from a pressurized test tube to avoid gas bubble formation. Samples were collected from both outlets using 6-port 2-way valves, with sample loops of a tubing length corresponding to 100 µL, placed in the fluid path between each chip outlet and the corresponding syringe. The flow rates and flow velocities for all experiments are given in Supplementary Table 1.

### GAF characterization using beads

An aqueous solution containing 5 w/v % Ficoll PM70 (GE Healthcare) was prepared as high impedance buffer. 2 g/L BSA (Sigma-Aldrich) was added as blocking agent to all the solutions to avoid particles sticking to the channel walls. As sample input, a mix of different spherical fluorescent particles, ranging from 0.2 to 3 µm was prepared. The beads with a nominal diameter of 0.2, 0.5, 1.0 and 2.0 µm were obtained from the flow cytometry sub-micron size reference kit (2% Solids Ref F8888, Thermo Fisher Scientific). 3-µm-beads were obtained from Sigma Aldrich. The bead concentration was 10^6^ beads/mL for each size of bead.

The output tubes (BD 5 mL round-bottom Falcon tube) were weighed before and after acquiring the sample to determine the volume collected in each tube. 50 µL of a stock solution of 840-nm red fluorescent beads with known concentration (1.71 × 10^5^/mL) were added to 50 µL of the acquired samples before quantifying the sample in the FACS Canto (BD). This allowed the determination of the bead concentration in the acquired samples. This enabled determination of the total amount of beads collected in the center outlet and side outlet.

### Bacterial culturing conditions

For acoustophoresis experiments *E. coli* (ATCC 11775) were grown in Tryptic soy broth (TSB, Becton Dickinson) with 0.01% CaCl, 0.16% BSA and 10% glycerol overnight at 37 °C. *S. pneumoniae* TIGR4 (BHN 842) and *S. aureus* (ATCC 25923) were incubated on Columbia blood agar plates overnight at 37 °C and 5% CO_2_. Suspension cultures of the gram-positive bacteria were prepared in Todd Hewitt medium with 0.5% yeast extract (THY, BD) and incubated at 37 °C in ambient atmosphere. Bacteria were diluted in isotonic NaCl solution containing 5% TSB, stored on ice and treated with 20 µg/mL tetracycline (Sigma) to prevent growth. For viability determination in lysates, all species were grown over night on Columbia blood agar plates at 37 °C and 5% CO_2_ from where a suspension culture in THY medium was started*. S. pneumoniae* suspension culture was incubated at 37 °C in ambient atmosphere whereas *E. coli* and *S. aureus* cultures were grown with additional shaking (~200 rpm).

### GAF of bacteria

Characterization of bacteria in the acoustophoresis set-up was performed in water with 2 g/L BSA, with 10^4^–10^5^ colony forming units/mL bacteria and a similar amount of 1 µm polystyrene beads which used for comparison with bead experiments. Tetracycline treated bacteria (10^5^–10^6^ colony forming units/mL) were stained in water containing 2 g/L BSA with 50 µM Syto9 (Invitrogen) for 15 minutes at 37 °C. Bacteria were spiked into lysates that were undiluted (corresponding to 13,3% hematocrit of an original 40% hematocrit blood sample) or 3× diluted with water containing 2 g/L BSA (corresponding to 4,4% hematocrit of an original 40% hematocrit blood sample). Bacteria, beads and debris were quantified in the FACS Canto (BD) using Trucount tubes (BD).

### Blood samples

Blood samples were collected from anonymous healthy adult volunteers at Skåne University hospital, Lund, Sweden, and at Blodcentralen at Karolinska University Hospital, Stockholm, Sweden, with buffered sodium citrate as anticoagulant (BD Vacutainer, 0.109 M). For acoustofluidic experiments, the hematocrit was determined using the Hematocrit 210 (Hettich, Germany) and Micro hematocrit tubes (ISO 12772, Brand GmbH &Co KG) according to the manufacturer’s instructions. The sample hematocrit was adjusted to 40% with red blood cell concentrate or plasma from the same blood obtained by centrifugation at 2000 × g for 10 minutes. The blood was used on the same day of withdrawal for lysis buffer characterization and within 36 hours for acoustofluidic experiments. All methods were carried out in accordance with relevant guidelines and regulations. According to the Regional Ethical Review Board at Lund University, no ethical approval was required in that case. Due to the anonymization, we cannot provide informed consent statements from the blood donors. The healthy volunteers gave their consent for the donation orally to the health care staff taking the blood samples according to local practice.

### Lysis buffer composition, lysis and viability determination

Lysis buffer contained 30 mg/mL saponin from Quillaja bark (Sigma Aldrich), 3 mg/mL tetradecyl sulfate sodium salt (Sigma Aldrich)^75^ and 10 mg/mL choline chloride (Sigma Aldrich) conjointly dissolved in water and complemented with 3 mM menadione (Sigma Aldrich)^76^ dissolved in DMSO (with a final concentration of 3% in the lysis buffer). One part lysis buffer was added to two parts of blood and incubated at 37 °C for 25 minutes. Thereafter an equal amount of water was added to the lysate^44^ and incubation was continued for 5 minutes. Lysis was stopped through a 40 times dilution of the blood volume (1 part) with 0.01 M PBS + 2 g/L BSA (lysis agents and diluent = 39 parts) and samples were characterized using the Gallios Flow Cytometer (Beckmann Coulter). Each sample was analyzed in two different dilutions (using PBS + 2 g/L BSA as solvent) and cytometer settings allowing for the recording of blood cells as well as smaller sized debris. For blood cells the gates determined by whole blood for red and white blood cells as well as platelets were used to determine the amount of similarly sized particles after lysis. For the counting of small particles and the estimation of the size equivalent of the debris, cytometer settings were defined using Megamix Plus FSC beadmix (Biocytex). Quantification was performed using Trucount tubes (BD) and data was analyzed with the Kaluza Analysis Software (v. 1.3, Beckmann Coulter, https://www.mybeckman.se/flow-cytometry/software/kaluza).

For the determination of bacterial viability 10^5^ cfu/mL were spiked in whole blood and treated with the lysis buffer according to the above described procedure as well as PBS as control treatment. To determine bacterial input concentration bacteria were spiked in THY and treated with twice the THY parts of PBS and incubated for 30 minutes. Lysis was stopped through diluting the blood volume 400 times with THY medium in total, bacteria were plated on blood agar plates and colonies were counted after overnight incubation. The presented results are triplicate treatments of blood from one day.

### Hemoglobin measurements

100 µL of the center and side outlet samples was directly pipetted into Greiner Cell Star Flat Bottom Chimeywell, clear, non-binding REF 6559 and absorption was measured at 540 nm (FLUOstar Omega, BMG Labtech).

## Data Availability

All data and computer code generated during the current study is available from the corresponding authors on request.

## Supporting Information

Supporting Information.
Supporting Information2.
Supporting Information3.
