# Supplementary material for: Gradient acoustic focusing of sub-micron particles for separation of bacteria from blood lysate
Source: Sci Rep. 2020 Feb 28;10:3670. doi: 10.1038/s41598-020-60338-2 (PMC7048738; doi:10.1038/s41598-020-60338-2)
Supplement: Supplementary file 1 — Supporting Information. [file 41598_2020_60338_MOESM1_ESM.pdf]

Supplementary Information for

Gradient acoustic focusing of sub-micron particles for separation of bacteria from blood lysate

David Van Assche+, Elisabeth Reithuber+, Wei Qiu, Thomas Laurell, Birgitta Henriques-Normark, Peter Mellroth, Pelle Ohlsson* and Per Augustsson*

(+ Equal contribution)

*Corresponding authors:

Pelle Ohlsson, Department of Biomedical Engineering, Lund University, Ole Römers väg 3, 22363 Lund, Sweden, +46 46 222 7503, pelle.ohlsson@bme.lth.se, ORCID: 0000-0003-4038-1605

Per Augustsson, Department of Biomedical Engineering, Lund University, Ole Römers väg 3, 22363 Lund, Sweden, +46 46 222 9371, per.augustsson@bme.lth.se, ORCID: 0000-0001-8542-7924

Supplementary Materials and Methods

Electrical resistance volumetric sizing of bacteria

Bacteria diluted in isotonic NaCl solution containing 5 % trypticase soy broth (Becton Dickinson) stored on ice and treated with 20 µg/mL tetracycline (VWR) were measured in triplicates using a Multisizer 3 Coulter Counter (Beckmann Coulter) with a 30 µm aperture tube.

Density centrifugation measurement

Pelleted bacteria (from 3 mL of the above described culture spun at 10 000 x g for 5 minutes) were dissolved in Optiprep density gradient medium (Sigma) diluted in phosphate-buffered saline (PBS) in 2% incrementing steps, serving as a known standard whose density was determined with a Density and Sound Velocity Meter DSA 5000M (Anton Paar, Malmö, Sweden), **Supplementary Fig. 4**. Bacteria in the density medium were centrifuged at 10 000 x g for 5 minutes and the density range was assessed by eye using the sink/float principle.

Speed of sound measurements

The measurements of speed of sound were performed on a Density and Sound Velocity Meter (DSA 5000M, Anton-Paar).

Viscosity measurements

Viscosities of Ficoll PM70 solutions were carried out on a viscometer (MINIVIS II, Grabner instruments, Vienna, Austria)

Lysis buffer optimization

Lysis buffer composition:

All components in the following section were purchased from Sigma Aldrich, except Trypticase soy broth (TSB) that was from Becton Dickinson, glucose from Fluka, DMSO from Life technologies and proteinase K from Qiagen.

Lysis buffer 1 (LB 1) was adapted from low Triton buffer #3 from Kirchner *et. al* [1] and contains 0.7 g/L L-cysteine HCl, 0.3 g/L sodium thioglycolate, 2.6 g/L (0.26% (w/v)) saponin from quillaja bark, 2.5 g/L glucose and 0.03% (v/v) Triton-X 100. LB 2 was composed as lysis buffer 1 but with 7 g/L L-cysteine HCl.

Saponin from quillaja bark was included with 10 g/L (1% (w/v)) in LB 4 and 10 and with 30 g/L (3% (w/v)) to buffers LB 5-9 and LB 11 [2, 3]. LB 6, 9 and 11 contained 3 g/L (0.3% (w/v)) tetradecyl sulfate sodium salt (STS, Sclerosants) [4] and LB 7 contained 4 g/L (0.4% (w/v)) thereof. 2-methyl-1,4-naphtoquinone (Menadione, Vitamin K3) dissolved in di-methyl sulfoxid (DMSO) was supplied from a 17.2 g/L stock solution to a final concentration of 0.52 g/L (3 mM) in lysis buffer 8-11 [5]. 10.4 g/L (74 mM) choline chloride was conjointly dissolved with the other ingredients in lysis buffer 4 to 11.

To establish hypertonic conditions 292.2 g/L (5M) sodium chloride was included in LB 10 [6]. Additional enzymatic treatment was employed in LB 11 with 10 g/L lipase from porcine pancreas dissolved in the buffer followed by a dilution of proteinase K to a final concentration of 3 g/L from the stock solution (20 mg/mL) during incubation.

Components in LB1 and 2 were dissolved in TSB, whereas components in LB4-11 were dissolved in water. All lysis buffers were filtrated (0.2 µm) before use.

Lysis procedure:

In detail, the following lysis procedures were carried out in which volumes were scaled up according to the experimental needs: For lysis buffer 1 and 2 100 µL of lysis buffer were added to 50 µL of blood and incubated for 30 minutes at 37 degrees (Procedure 1, Supplementary Table 7). For lysis buffer 3-10 25 µL of lysis buffer were added to 50 µL of blood and incubated for 25 minutes, thereafter 75 µL of PBS for lysis buffer 3 (Procedure 2, Supplementary Table 7) and water for lysis buffer 4-10 [3]

(Procedure 3, Supplementary Table 7) were added to the sample and incubation was continued for 5 minutes. Incubation with the lipase-containing lysis buffer 11 was carried out for 20 minutes with volume ratios as in lysis procedure 2&3 where after an equal amount of water and 25 µL proteinase K were added and incubation continued for 10 minutes at 37 °C (Procedure 4, Supplementary Table 7). The volume-increase through proteinase addition was taken into account when diluting the sample 40 times with PBS (1 part blood, 39 parts lysis agents and PBS) to stop the lysis procedure. Flow cytometry analysis was carried out as described in the main manuscript. Whole blood (WB) was treated along lysis procedure 2 (Supplementary Table 7), with the exception that lysis buffer was replaced by PBS.

Determination of bacterial viability was carried out as described in the manuscript and to determine the input concentration bacteria were spiked into THY medium and treated with PBS according to lysis procedure 1.

In supplementary table 7 flow cytometry data from three independently prepared lysis buffers that were tested on blood from one donor are shown. Viability evaluation results of triplicate treatments on blood from one day are shown, which are repeatable with blood from another day.

Supplementary Note 1: Simulated particle trajectories

Particles suspended in a microchannel (length L = 27 mm, width W = 375 μm, height H = 150 μm) with half-wave standing-wave field primarily experience acoustic radiation force $\boldsymbol{F}_{\mathrm{rad}}$ and Stokes drag force $\boldsymbol{F}_{\mathrm{drag}}$ induced by acoustic streaming and Poiseuille flow. In this well-established system, analytical solution for $\boldsymbol{F}_{\mathrm{rad}}$ has been obtained to be[7]

$$F_{rad} = 4k_0\pi a^3 E_{ac}\Phi\sin(2k_0 y), \quad (1)$$

where k_0, a, E_{ac}, Φ are wave number, particle radius, acoustic energy density, and acoustic contrast factor, respectively. Here, Φ is calculated as

$$\Phi = \frac{1}{3}f_1 + \frac{1}{2}Re(f_2), \quad (2a)$$

$$f_1 = 1 - \tilde{\kappa}, \quad (2b)$$

$$f_2 = \frac{2[1-\Gamma](\tilde{\rho}-1)}{2\tilde{\rho}+1-3\Gamma}, \quad (2c)$$

$$\Gamma = -\frac{3}{2}\left[1 + i\left(1+\tilde{\delta}\right)\right]\tilde{\delta}, \quad (2d)$$

where $\tilde{\kappa}$, $\tilde{\rho}$, and $\tilde{\delta}$ are the ratio of particle compressibility κ_p = 249 TPa^{-1} to the compressibility of the medium κ_m = 440.5 TPa^{-1}, the ratio of the particle density ρ_p = 1050 kg m^{-3} to the medium density ρ_m = 1005.6 kg m^{-3}, and the ratio of the thickness of viscous boundary layer δ = 0.4 μm to a. The time-averaged streaming velocity has been solved analytically by Lord Rayleigh[8] for the two components in the y-z cross-section.

$$\langle v_{2y}\rangle = \frac{3}{8}\frac{{v_{ac}}^2}{c_m}\sin\left[2k_0\left(y+\frac{W}{2}\right)\right]\left[1-3\frac{z^2}{(H/2)^2}\right]\frac{1}{2} \quad (3)$$

And

$$\langle v_{2z}\rangle = \frac{3}{8}\frac{{v_{ac}}^2}{c_m}k_0 H\sin\left[2k_0\left(y+\frac{W}{2}\right)\right]\left[\frac{z^3}{(H/2)^3}-\frac{z}{H/2}\right]\frac{1}{2}, \quad (4)$$

where v_{ac} is the amplitude of first-order velocity field $\boldsymbol{v}_1$. The Poiseuille flow in a rectangular cross-section microchannel has no analytical solution, but a Fourier sum can be derived,[9]

$$v_x(y,z) = \frac{4H^2\Delta p}{\pi^3\eta L}\sum_{n,odd}^{\infty}\frac{1}{n^3}\left[1-\frac{\cosh\left(n\pi\frac{y}{H}\right)}{\cosh\left(n\pi\frac{W}{2H}\right)}\right]\sin\left(n\pi\frac{z}{H}\right), \quad (5)$$

where η = 1.17 mPa s and Δp are the medium viscosity and the constant pressure difference over the channel. The relation between the volumetric flow rate Q and Δp is described as

$$Q = \frac{8H^3W\Delta p}{\pi^4\eta L}\sum_{n,odd}^{\infty}\left[\frac{1}{n^4}-\frac{2H}{\pi W}\frac{1}{n^5}\tanh\left(n\pi\frac{W}{2H}\right)\right]. \quad (6)$$

In this simulation, $\boldsymbol{F}_{\mathrm{rad}}$, $\boldsymbol{F}_{\mathrm{drag}}$ induced by acoustic streaming and Poiseuille flow, and the gravity and buoyance forces of the particle $\boldsymbol{F}_{\mathrm{g}}$ and $\boldsymbol{F}_{\mathrm{buo}}$ are all included. No inertia effect is taken into account due to the small particle mass and low Reynolds number regime in our system. Following the experimental conditions, E_{ac} and Q are set to 250 J m^{-3} and 28 μL min^{-1}, respectively. The fourth-order Runge-Kutta method is used to compute the particle trajectories with a time interval of 0.1 s. In the simulation of inhomogeneous medium, acoustic streaming is set to zero due to the stabilizing acoustic body force that suppresses streaming within the time-scale in this study.[10]

Supplementary Note 2: Calculation diffusion constant Ficoll PM70.

Stokes-Einstein equation to calculate the diffusion constant:

$$D = \frac{k_B T}{6\pi\eta r} = 2.7 \cdot 10^{-11}\ m^2/s \tag{7}$$

with,

k_B Boltzmann constant = $1.38.10^{-23}$ J/K

T = 298 K

r, Stokes' radius Ficoll PM70 = 5.1 nm [11]

η, viscosity = 1.586 mPas

Supplementary Note 3: Lysis buffer evaluation

In bacteremic samples bacteria are rare events when compared to the abundant blood cells, as sometimes only 10-100 colony forming units (cfu) are reported in a milliliter of a bacteremic blood sample [12]. For label free particle separation based on size it is important to consider that bacteria and blood cells may be associated to each other to an unknown extent and therefore might hinder discriminative separation.

We therefore believe that selective blood cell lysis as a first step in sample preparation could be beneficial for label free downstream operations in a sepsis diagnosis perspective. This is supported by the beneficial effects reported for the lysis centrifugation method [2].

Hence we measured the suitability of published lysis buffers in terms of the efficiency of red and white blood cell lysis, abundance of remaining platelet sized particles as well as particles of common bacteria size (~1 µm) and monitored the debris below that size (≤0.5 µm) that was produced during lysis. Furthermore we examined conditions specifically designed to achieve the reduction of these small particles and the results for a representative buffer selection are summarized in supplementary Table 7.

Our results revealed that most buffers were similarly effective in achieving red and white blood cell lysis with a decrease in cell counts of about 4 orders of magnitude in the comparative analysis where lysis time was standardized to 30 minutes. Platelet-sized particles were over all reduced by 1-2 orders of magnitude and particles of common bacterial size were reduced about 0.5-1 order of magnitude after applying treatment with lysis buffers (LB). The amount of smaller sized debris (≤0.5 µm particles) increased with about 1 order of magnitude for most buffers (except LB 11). Importantly, the viability of the common sepsis causing gram positive *Staphylococcus aureus*, *Streptococcus pneumoniae* and the gram negative *Escherichia coli* was well retained for most tested lysis buffers (Supplementary Table 7).

All lysis buffers presented in Supplementary Table 7 contain saponin in varying concentrations. This glycoside from quillaja bark permeabilizes membranes primarily in a cholesterol dependent manner, with higher potency towards eukaryotic compared to prokaryotic cell membranes [13, 14, 15] making it suitable for selective lysis purposes.

Most lysis buffers contain choline chloride which can inhibit the suicidal autolysis that the sepsis causing pathogen *Streptococcus pneumoniae* is prone to undergo when challenged with detergents [16]. With a final concentration of 25 mM it is above the minimal required concentration that was reported to be inhibitive to pneumococcal autolysis for the lysis-centrifugation method [17].

As reported by Kirchner et. al. [1] the buffer LB1 is effectively reducing blood cells without too much debris production while retaining bacterial viability. Since especially gram-positive bacteria are less susceptible to reducing agents [18] we increased the L-cysteine HCl concentration in LB2 without the aspired decrease of debris size.

The cell wall protects prokaryotes from osmotic stress, which can therefore be employed to selectively lyse the more susceptible eukaryotic cells. However, the hypertonic conditions [6] we tried to establish in LB10 did yield worse blood cell lysis compared to other buffers tested. Reported hypotonic conditions [3] were established when employing lysis procedure 3. In the tested setup, no measurable effect could be recorded when directly comparing LB3 and LB5.

To enhance platelet lysis we introduced tetradecyl sulfate sodium salt a detergent sclerosant in two concentrations that were reported to achieve around 50 % platelet lysis in the corresponding amount of whole blood [4]. Due to the effects on bacterial viability 0.15 % final tetradecyl sulfate sodium salt concentration in the lysate was preferred and further employed in LB9. Menadione or Vitamin K_3 was reported to result in platelet lysis [5] and was therefore employed to 1mM final concentration in LB8 and LB9, whereas the standardized incubation time of 30 minutes might not allow the full desired effect.

LB11 contained lipase and was followed by a proteinase incubation step with the intended enhancement of blood cell lysis and further debris reduction. The enzymatic treatment was effective, however not favorable for pneumococcal viability.

To allow comparative analysis of the lysis buffers the duration of lysis was set for 30 minutes after which lysis was stopped through an extensive dilution of buffer components using PBS (Supplementary Table 7). Despite the presumably high enough plasma protein concentration to avoid absorbance of particles to test tube walls [19] this resulted in a lower particle count compared to when PBS with 2 g/L BSA was used for stopping the lysis procedure by copious dilution (Figure 5). Nonetheless, Supplementary Table 7 allows comparative analysis of the lysis buffers and LB9 treated in both ways (Figure 5, Supplementary table 7) can serve as a landmark to compare the influence of BSA in the dilution buffer. To conclude, among the conditions tested there were some fulfilling the criteria of efficient blood cell lysis with as small sized as possible debris production while retaining bacterial viability. We could not find a lysis buffer that would meet all criteria exceptionally well. For application in the GAP experiments, we chose to lyse the blood with LB9.

Supplementary Fig. 1: Acoustic radiation force on particles and acoustic streaming.

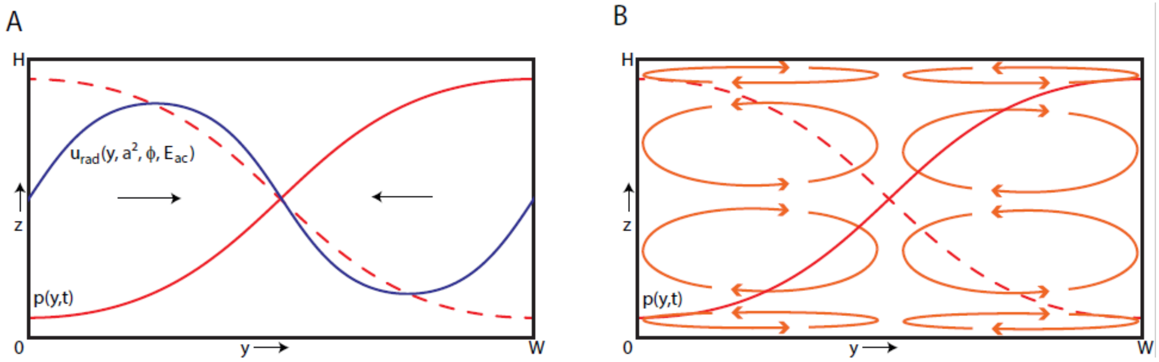

(A) Pressure field (p(y,t)) scatters on suspended objects resulting in an acoustic radiation force and an acoustic-radiation-induced velocity u_{rad} towards the pressure minimum. **(B)** Acoustic streaming, which originates from boundary layer effects, exerts a drag force on objects. The streaming initiates in the top and bottom boundary layer (drawn here but not to scale) and recirculates in the bulk. This results in a drag force towards the sides of the channel at the top and bottom, while at mid-height the drag force points towards the center of the channel width.

Supplementary Fig. 2: Central fraction of particles in homogenous medium over time using stop flow.

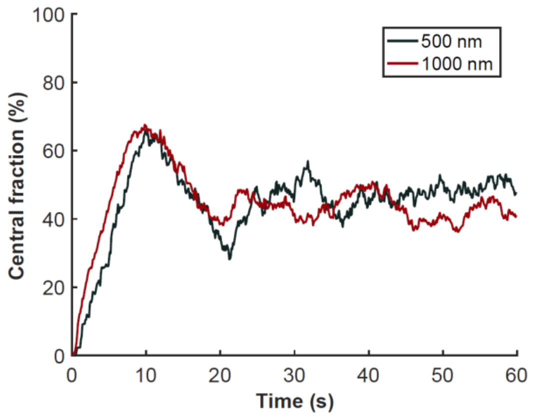

Quantification of 500-nm and 1000-nm-particles obtained in the central fraction over time by operating the device with stop flow in homogenous medium.

Supplementary Fig. 3: Size distributions of investigated bacterial species compared to 1000-nm-particles.

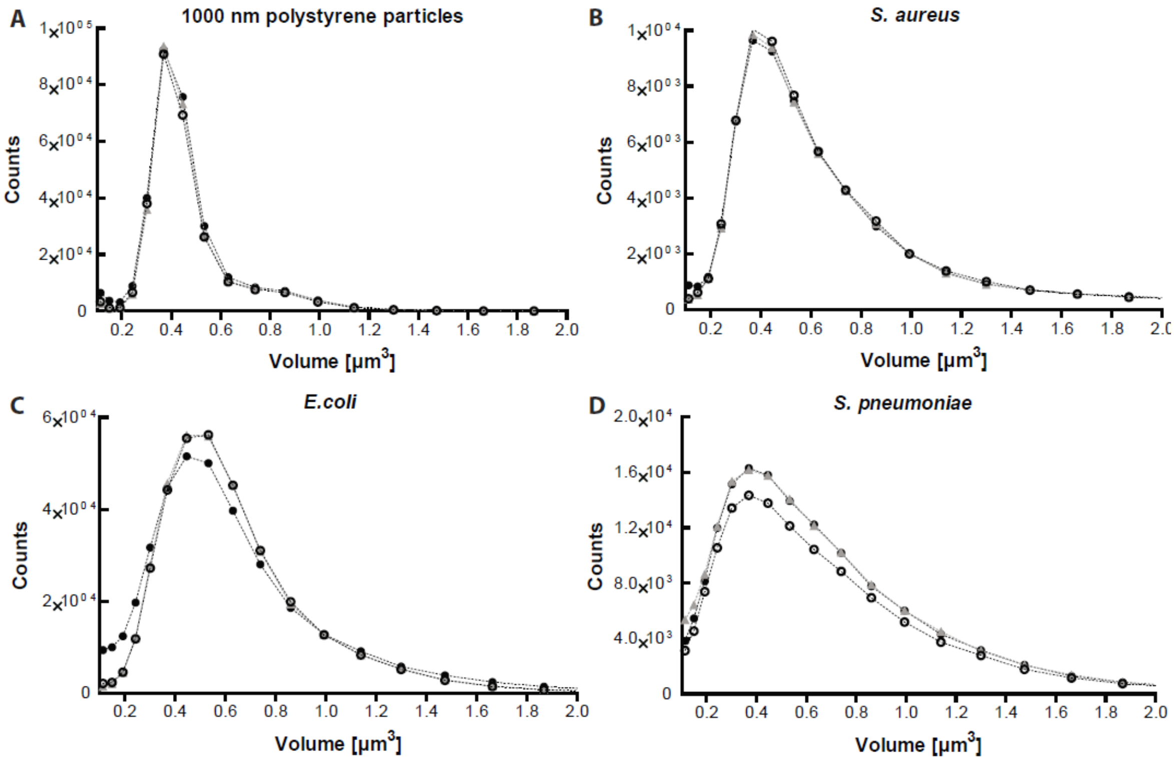

Spherical volumes calculated from diameters obtained from Coulter Counter (BC) measurements of **(A)** 1000 nm particles, **(B)** *Staphylococcus aureus*, **(C)** *Escherichia coli* and **(D)** *Streptococcus pneumoniae*. The assumption of a spherical bacterial shape introduces an error regarding the actual bacterial morphology, allows however estimation of size distribution. N=3.

Supplementary Fig. 4: **Standard curve for bacterial density estimation.**

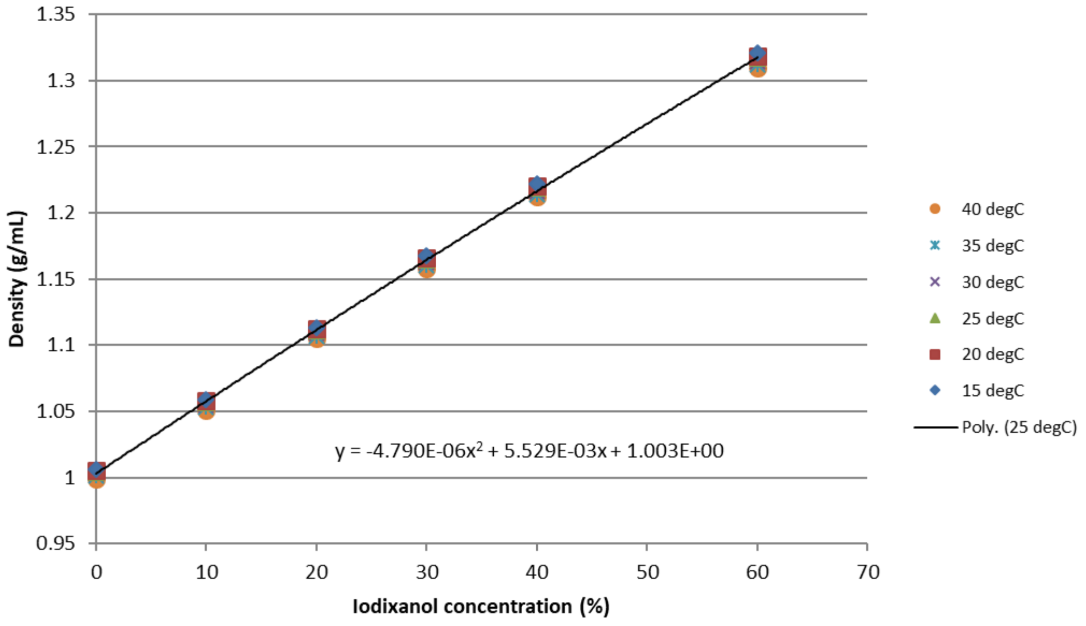

Iodixanol concentration (OptiPrep, Sigma Aldrich) and its corresponding density at varying temperatures measured with a Density and Sound Velocity Meter (DSA 5000M, Anton-Paar).

Supplementary Fig. 5: Flow cytometry protocol setup and particle counting principle.

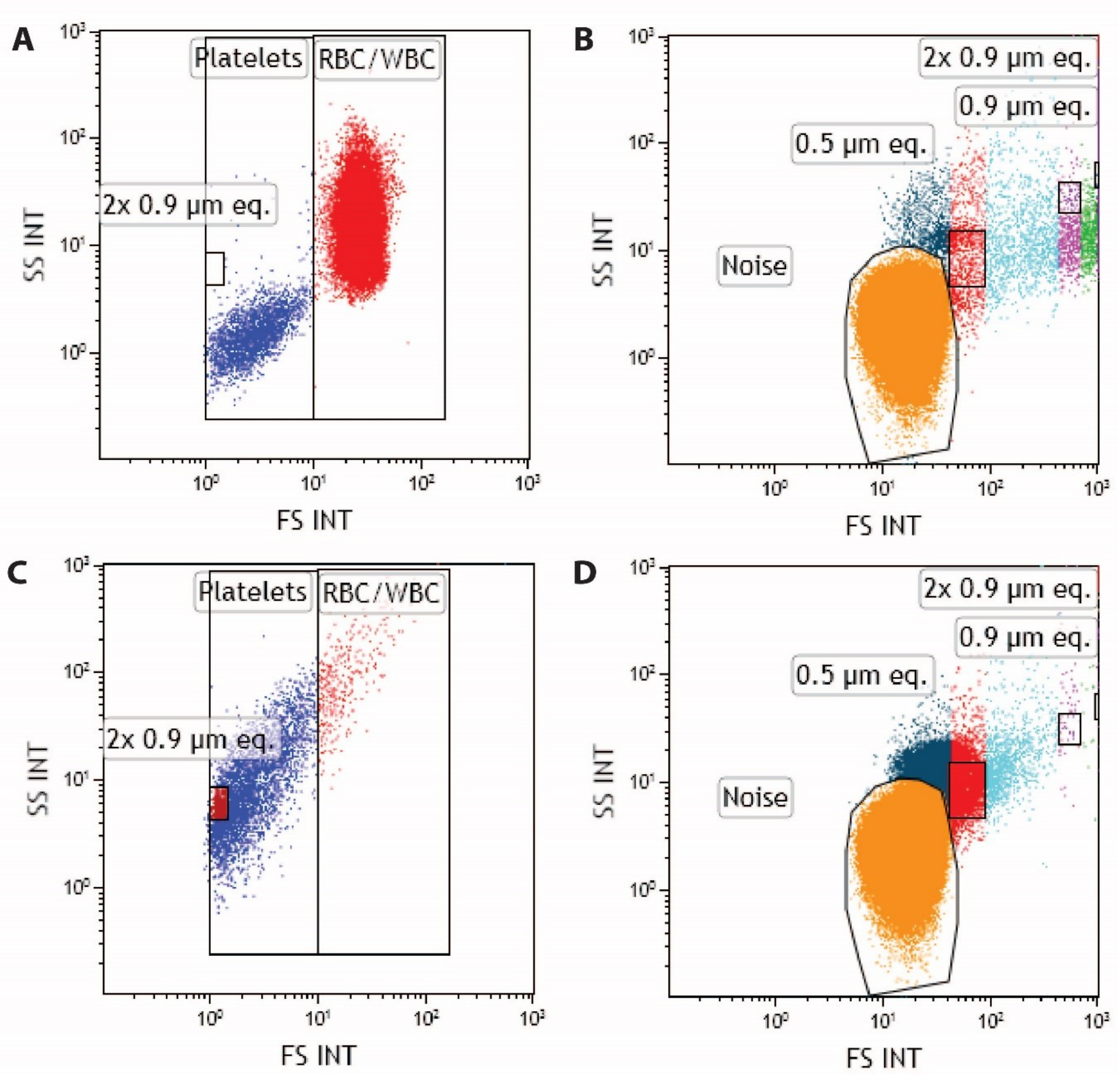

Whole blood **(A, B)** and lysate **(C, D)** were analyzed with settings allowing the counting of large cells like red blood cells (RBC) and white blood cells (WBC) as well as platelets **(A-C)** and settings for the counting of sub-micron particles **(B-D)**. In order to allow for a size estimation and quantification of small particles, cytometer settings were defined using the Megamix Plus FSC beadmix (Biocytex). Data was analyzed with the Kaluza Analysis Software (Beckmann Coulter).

Supplementary Table 1: Flow settings at the chip in- and outlets and composition of the suspensions supplied in the central and side inlet for the experiments displayed in the indicated figures

Figure	Flow rate	Mean flow velocity	Central inlet suspension	Side inlet suspension
**Fig. 1D**	Stopped-flow	0 m/s		
**Fig. 1F**	Stopped-flow	0 m/s		
**Fig. 1I**	Center in = 16 µL/min<br>Side in = 12 µL/min<br>Center out = 14 µL/min<br>Side out = 14 µL/min	Center in = 4.7 mm/s<br>Side in = 3.6 mm/s<br>Center out = 4.1 m/s<br>Side out = 4.1 m/s	BSA (2 g/L)	BSA (2g/L) + bead mix
**Fig. 1J**	Center in = 16 µL/min<br>Side in = 12 µL/min<br>Center out = 14 µL/min<br>Side out = 14 µL/min	Center in = 4.7 mm/s<br>Side in = 3.6 mm/s<br>Center out = 4.1 m/s<br>Side out = 4.1 m/s	Ficoll PM70 (5 w/v%) + BSA (2g/L)	BSA (2g/L) + bead mix
**Fig. 2A**	Center in = 16 µL/min<br>Side in = 12 µL/min<br>Center out = 14 µL/min<br>Side out = 14 µL/min	Center in = 4.7 mm/s<br>Side in = 3.6 mm/s<br>Center out = 4.1 m/s<br>Side out = 4.1 m/s	Varying.<br>For ΔZ = -0.25: BSA (2 g/L)<br>For ΔZ = 0: BSA (2 g/L)<br>For ΔZ = +0.04: Ficoll PM70 (0.125 w/v%) + BSA (2 g/L)<br>For ΔZ = +0.13: Ficoll PM70 (0.25 w/v%) + BSA (2 g/L)<br>For ΔZ = +0.25: Ficoll PM70 (0.5 w/v%) + BSA (2 g/L)<br>For ΔZ = +0.53: Ficoll PM70 (1 w/v%) + BSA (2 g/L)<br>For ΔZ = +1.08: Ficoll PM70 (2 w/v%) + BSA (2 g/L)<br>For ΔZ = +2.76: Ficoll PM70 (5 w/v%) + BSA (2 g/L)	Varying.<br>For ΔZ = -0.25: Ficoll PM70 (0.5 w/v%) + BSA (2 g/L) + bead mix.<br>For others: BSA (2 g/L) + bead mix
**Fig. 3A**	Varying. Total flow of 10 µL/min, 20 µL/min, 30 µL/min, 50 µL/min, 100 µL/min, 150 µL/min and 200 µL/min.	Varying. Total flow of 3.0 mm/s, 5.9 mm/s, 8.9 mm/s, 15 mm/s, 30 mm/s, 44 mm/s and 59 mm/s.	Ficoll PM70 (5 w / v %) + BSA (2 g/L)	BSA (2 g/L) + bead mix
**Fig. 4**	Center in = 16 µL/min<br>Side in = 12 µL/min<br>Center out = 14 µL/min<br>Side out = 14 µL/min	Center in = 4.7 mm/s<br>Side in = 3.6 mm/s<br>Center out = 4.1 m/s<br>Side out = 4.1 m/s	Ficoll PM70 (5 w/v%) + BSA (2 g/L)	BSA (2 g/L) + *S. aureus*, *E. coli* or *S. pneumoniae* + 1000 nm beads
**Fig. 5A**	Center in = 16 µL/min<br>Side in = 12 µL/min<br>Center out = 14 µL/min<br>Side out = 14 µL/min	Center in = 4.7 mm/s<br>Side in = 3.6 mm/s<br>Center out = 4.1 m/s<br>Side out = 4.1 m/s	Varying.<br>Ficoll PM70 (2 w/v%) + BSA (2 g/L) for bacteria in water<br>Ficoll PM70 (4 w/v%) + BSA (2 g/L) for 3x diluted lysate<br>Ficoll PM70 (8 w/v%) + BSA (2 g/L) for undiluted lysate	Varying.<br>BSA (2 g/L) + *S. aureus* + 1000 nm beads<br>3x diluted lysate + *S. aureus* + 1000 nm beads<br>Undiluted lysate + *S. aureus* + 1000 nm beads
**Fig. 5B**	Center in = 16 µL/min<br>Side in = 12 µL/min<br>Center out = 14 µL/min<br>Side out = 14 µL/min	Center in = 4.7 mm/s<br>Side in = 3.6 mm/s<br>Center out = 4.1 m/s<br>Side out = 4.1 m/s	Ficoll PM70 (4 w/v%) + BSA (2 g/L)	3x diluted lysate + *S. aureus* + 1000 nm beads

Supplementary Table 2: Bead recoveries* for experiments shown in Figure 1I (homogenous medium) and 1J (inhomogenous medium).

	200 nm	500 nm beads	1000 nm beads	2000 nm beads	3000 nm beads	All bead sizes
**Homogeneous medium**	82.0 ± 3.8 %	84.8 ± 4.0 %	87.4 ± 4.7 %	80.0 ± 7.0 %	63.6 ± 11.3 %	79.6 ± 10.8 %
**Inhomogeneous medium**	71.7 ± 7.7 %	76.2 ± 8.2 %	78.1 ± 10.4 %	77.6 ± 9.5 %	82.6 ± 10.5 %	77.2 ± 10.0 %
**Average**	75.4 ± 8.2 %	79.3 ± 8.1 %	81.5 ± 9.8 %	78.5 ± 8.8 %	75.8 ± 14.1 %	78.1 ± 10.3%

* Recoveries are calculated as the total beads collected through both outlets relative to the input before administration to the microfluidic device. The values are adjusted for the anticipated internal loss in the setup attributed to its swept volume, limiting the maximum possible recovery to 82%.

Supplementary Table 3: Bead recoveries* for experiments shown in Figure 2A.

	200 nm beads	500 nm beads	1000 nm beads	2000 nm beads	3000 nm beads	All bead sizes
**ΔZ = - 0.25 %**	81.2 ± 1.3 %	80.6 ± 0.7 %	68.7 ± 1.6 %	64.8 ± 3.8 %	52.6 ± 6.9 %	69.6 ± 11.3 %
**ΔZ = 0.00%**	83.5 ± 1.3 %	83.3 ± 1.7 %	90.2 ± 3.9 %	81.8 ± 6.4 %	61.2 ± 16.6 %	80.0 ± 12.8 %
**ΔZ = + 0.04%**	82.1 ± 0.2 %	85.2 ± 3.5 %	83.0 ± 1.8 %	68.5 ± 1.7 %	65.3 ± 5.3 %	76.8 ± 8.8 %
**ΔZ = + 0.13%**	83.9 ± 5.1 %	88.0 ± 8.6 %	85.4 ± 5.1 %	70.1 ± 7.2 %	60.8 ± 11.8 %	77.6 ± 13.2 %
**ΔZ = + 0.25%**	83.9 ± 5.1 %	88.0 ± 8.6 %	85.4 ± 5.1 %	70.1 ± 7.2 %	60.8 ± 11.8 %	77.6 ± 13.2 %
**ΔZ = + 0.53%**	84.8 ± 2.6 %	84.8 ± 4.1 %	81.7 ± 1.9 %	74.1 ± 1.0 %	66.8 ± 3.5 %	74.6 ± 8.7 %
**ΔZ = + 1.08%**	69.8 ± 2.2 %	76.4 ± 2.6 %	74.5 ± 7.3 %	59.9 ± 4.4 %	59.1 ± 3.8 %	68.0 ± 8.5 %
**ΔZ = + 2.76%**	69.5 ± 3.4 %	70.5 ± 4.4 %	84.9 ± 3.0 %	73.9 ± 5.2 %	80.2 ± 5.2 %	75.8 ± 7.3 %
**Average**	79.0 ± 6.6 %	81.1 ± 6.7 %	81.4 ± 7.4 %	70.7 ± 7.8 %	63.4 ± 11.2 %	75.1 ± 10.8 %

* Recoveries are calculated as the total beads collected through both outlets relative to the input before administration to the microfluidic device. The values are adjusted for the anticipated internal loss in the setup attributed to its swept volume, limiting the maximum possible recovery to 82%.

Supplementary Table 4: Bead and bacteria recoveries* for experiments shown in Figure 4A

	**200 nm beads**	**500 nm beads**	***S. pneumoniae***	***E. coli***	***S. aureus***	**1000 nm beads**	**2000 nm beads**	**3000 nm beads**
**Water + BSA (2g/L): 400 V^2**	62.9 ± 2.7%	71.6 ± 0.7%	91.7 ± 3.3%	83.9± 11.0%	94.9 ± 1.5%	89.5 ± 0.1%	65.7 ± 10.7%	64.8 ± 8.3%

* Recoveries are calculated as the total beads collected through both outlets relative to the input before administration to the microfluidic device. The values are adjusted for the anticipated internal loss in the setup attributed to its swept volume, limiting the maximum possible recovery to 82%.

Supplementary Table 5: Bead and bacteria recoveries* for experiments shown in Figure 4B

	**1000 nm beads**	***E.coli***	***S. pneumoniae***	***S. aureus***
**Water + BSA (2 g/L): 25 V^2**	70.11 ± 5.09 %	not determined	not determined	85.80 ± 16.95 %
**Water + BSA (2 g/L): 100 V^2**	77.93 ± 0.14 %	77.60 ± 11.13 %	84.32 ± 2.95 %	84.23 ± 5.83 %
**Water + BSA (2 g/L): 225 V^2**	84.02 ± 0.07 %	91.57 ± 4.00 %	90.50 ± 6.88 %	83.09 ± 11.73 %

* Recoveries are calculated as the total beads collected through both outlets relative to the input before administration to the microfluidic device. The values are adjusted for the anticipated internal loss in the setup attributed to its swept volume, limiting the maximum possible recovery to 82%.

Supplementary Table 6: Density of investigated bacterial species compared to beads measured by sink/float centrifugation

	**Density [g/cm³]**
**1 µm beads***	1.05
S. aureus	1.12 – 1.15
E.coli	1.08 – 1.14
S. pneumoniae	1.11 – 1.14

*From product manual FluoSpheres™ Size Kit #2 (Thermo Fisher Scientific)

Supplementary Table 7: Lysis protocol evaluation

	Lysis protocols	Lysis procedure	RBC/WBC-sized particles[a]	Platelet-sized particles[a]	Particles ~1 µm eq.[b]	Particles ≤ 0,5 µm eq.[c]	Viability[d]		
							S. aureus	*S. pneumoniae*	*E. coli*
**WB**		2	9.54 ± 0.07	8.21 ± 0.07	8.2 ± 0.06	8.12 ± 0.04	59.80 ± 8.29	95.76 ± 10.25	67.34 ± 11.38
**LB1**	**Low Triton buffer [1]**	1	5.40 ± 0.18	6.59 ± 0.03	7.30 ± 0.06	9.20 ± 0.01	77.40 ± 7.20	91.56 ± 16.16	81.82 ± 12.26
**LB2**	**Low Triton buffer, elevated L-Cysteine HCl**	1	5.79 ± 0.09	6.86 ± 0.02	7.42 ± 0.10	9.20 ± 0.02	85.58 ± 8.52	70.22 ± 9.51	52.12 ± 8.86
**LB3**	**3% Sap, 74mM Cc**	2	5.17 ± 0.07	6.57 ± 0.02	7.43 ± 0.06	9.20 ± 0.02	94.21 ± 25.31	80.43 ± 4.70	60.77 ± 2.84
**LB4**	**1% Sap, 74mM Cc**	3	5.63 ± 0.01	7.08 ± 0.11	7.47 ± 0.07	9.18 ± 0.03	73.68 ± 3.94	89.13 ± 25.04	47.59 ± 3.96
**LB5**	**3% Sap, 74mM Cc**	3	5.22 ± 0.04	6.61 ± 0.02	7.47 ± 0.07	9.19 ± 0.02	110.2 ± 8.54 *	98.4 ± 4.10 *	64.6 ± 9.41 *
**LB6**	**3% Sap, 74mM Cc, 0.3% STS**	3	5.13 ± 0.08	6.50 ± 0.01	7.53 ± 0.06	9.17 ± 0.02			
**LB7**	**3% Sap, 74mM Cc, 0.4% STS**	3	5.04 ± 0.01	6.44 ± 0.18	7.37 ± 0.09	9.12 ± 0.01	53.91 ± 8. 34	84.62 ± 15.68	63.2 ± 7.52
**LB8**	**3% Sap, 74mM Cc, 3mM Men**	3	5.75 ± 0.06	7.14 ± 0.004	7.56 ± 0.06	9.19 ± 0.02			
**LB9**	**3% Sap, 74mM Cc, 0.3% STS, 3mM Men**	3	5.42 ± 0.04	6.69 ± 0.18	7.75 ± 0.10	9.18 ± 0.03	91.4 ± 6.43 *	93.2 ± 2.05 *	68.3 ± 11.30 *
**LB10**	**1% Sap, 74mM Cc, 3mM Men, 5M NaCl**	3	6.51 ± 0.03	7.63 ± 0.08	7.52 ± 0.16	9.04 ± 0.10			
**LB11**	**LB 9 + enzymatic treatment**	4	5.04 ± 0.09	5.86 ± 0.22	7.13 ± 0.13	7.76 ± 0.20	93.75 ± 6.63	6.73 ± 9.52	62.34 ± 6.45

[a] Mean ± standard deviation (SD) of the decimal logarithm of particle counts per mL in the forward and side scatter gates which characterized the respective blood cells (red blood cells; white blood cells; platelets) in whole blood

[b] Mean (± SD) of the decimal logarithm of particle counts per mL with forward scatter characteristics ranging between the ones equivalent to 0.9 µm doublets and 0.5 µm particles of the Megamix plus FSC kit (BioCytex).

[c] Mean (± SD) of the decimal logarithm of particle counts per mL with a forward scatter equivalent and smaller than the one of 0.5 µm particles of the Megamix plus FSC kit (BioCytex) but clearly distinguishable from the noise.

[d] Percent viability (Mean (± SD)) of the respective bacteria spiked into whole blood (WB) and lysis buffer treated blood compared to average viability of bacteria incubated in nutrient broth

[*] Experiments performed on another day with corresponding viability in whole blood of 80 ± 4.93 % for *S. aureus*, 121.7 ± 11.34 % for *S. pneumoniae* and 53.1 ± 8.90 for *E.coli*.

Abbreviations: Sap – saponin, Cc – choline chloride, STS – sclerosant (tetradecyl sulfate sodium salt, Men – menadione (2-methyl-1,4-naphtoquinone) , RBC – red blood cells, WBC – white blood cells, WB – whole blood

Supplementary Table 8: Acoustic impedance of liquids at 25°C *

	Density (g/cm³)	Speed of sound (m/s)	Acoustic impedance [Pas/m]	Ficoll concentration for 1 % acoustic impedance difference (ΔZ = 1 %)
**Water + BSA (2 g/L)**	0.998 ± 0.5 E-06	1497.46 ± 0.004	1.5088E+06 ± 3.8	1.86%
**3x diluted lysate (4.4 % corresponding hematocrit)**	1.004 ± 0.7 E-06	1504.84 ± 0.01	1.5110E+06 ± 7.1	4.0 %
**Undiluted lysate (13.3 % corresponding hematocrit)**	1.017 ± 1.2 E-06	1520.76 ± 0.02	1.5468E+06 ± 15.8	8.49%

*One sample was analyzed per condition using 5 technical repeats.

Supplementary Table 9: Bead and bacteria recoveries* for experiments shown in Figure 6A

	**1000 nm beads**	***S. aureus***
**Water + BSA (2 g/L)**	92.38 ± 4.98 %	92.49 ± 2.72 %
**3x diluted lysate (4.4 % corresponding hematocrit)**	81.18 ± 3.36 %	79.77 ± 12.81 %
**Undiluted lysate (13.3 % corresponding hematocrit)**	74.15 ± 6.25 %	65.89 ± 7.71 %

* Recoveries are calculated as the ratio of the sample collected in both outlets and before administration to the microfluidic device. The values are adjusted for the anticipated internal loss in the setup attributed to its swept volume, limiting the maximum possible recovery to 82%.

Supplementary Table 10: Bead and bacteria recoveries* for experiments shown in Figure 6B

	**1000 nm beads**	***S. aureus***
**3x diluted lysate: 289 V^2**	91.68 ± 7.01 %	76.09 ± 1.57 %
**3x diluted lysate: 400 V^2**	81.18 ± 3.36 %	79.77 ± 12.81 %
**3x diluted lysate: 529 V^2**	75.84 ± 13.05 %	63.87 ± 21.71 %

* Recoveries are calculated as the ratio of the sample collected in both outlets and before administration to the microfluidic device. The values are adjusted for the anticipated internal loss in the setup attributed to its swept volume, limiting the maximum possible recovery to 82%.

Caption for Supplementary Movie 1

We performed particle tracking in 3D of 500-nm-diameter and 1-µm-diameter particles using General Defocusing Particle Tracking [20] for a 1 mm segment of the channel after temporarily stopping the flow. For a homogeneous system of liquids with matched acoustic impedances, the sound induces a steady acoustic streaming flow in the channel which leads to mixing. The evolution of the particle positions and the resulting fraction of particles in each channel segment show efficient mixing and similar behavior of both particles sizes.

Caption for Supplementary Movie 2

The same method as in Supplementary Movie S1 was employed. However, for an inhomogeneous system of liquids that form a gradient in acoustic impedance such that $\Delta Z > 0$, acoustic streaming rolls in the channel are suppressed and confined to the floor and ceiling of the channel. This enabled accumulation of 1-µm-diameter particles near the channel center, and separation of 1-µm-diameter particles from 500-nm-diameter particles.

Supplementary References

1. KIRCHER SM*, et al.* Formulations and process for isolating viable microorganism from positive blood cultures. https://patents.google.com/patent/WO2013130759A1 (2013).

2. TeKippe EM, Pence MA. Lysis-Centrifugation Methods of Blood Culture. In: *The Dark Art of Blood Cultures*). American Society of Microbiology (2017).

3. Zelenin S, Hansson J, Ardabili S, Ramachandraiah H, Brismar H, Russom A. Microfluidic-based isolation of bacteria from whole blood for sepsis diagnostics. *Biotechnology letters* **37**, 825-830 (2015).

4. Connor DE, Cooley-Andrade O, Goh WX, Ma DDF, Parsi K. Detergent Sclerosants are Deactivated and Consumed by Circulating Blood Cells. *Eur J Vasc Endovasc* **49**, 426-431 (2015).

5. Kim KA, Lee JY, Park KS, Kim MJ, Chung JH. Mechanism of menadione-induced cytotoxicity in rat platelets. *Toxicol Appl Pharm* **138**, 12-19 (1996).

6. Söderström. N. Hemolysis by Hypertonic Solutions of Neutral Salts. *Acta Physiologica Scandinavica* **7**, 56-68 (1944).

7. Barnkob R, Augustsson P, Laurell T, Bruus H. Measuring the local pressure amplitude in microchannel acoustophoresis. *Lab Chip* **10**, 563-570 (2010).

8. Rayleigh L. On the circulation of air observed in Kundt's tubes, and on some allied acoustical problems. *Philosophical Transactions of the Royal Society of London* **175**, 1-21 (1884).

9. Bruus H. *Theoretical Microfluidics*. Oxford University Press (2007).

10. Qiu W, Karlsen JT, Bruus H, Augustsson P. Experimental Characterization of Acoustic Streaming in Gradients of Density and Compressibility. *Physical Review Applied* **11**, 024018 (2019).

11. Ficoll PM70 Ficoll PM400: Data File 18-1158-27 AB, https://www.gelifesciences.co.jp/catalog/pdf/18115827.pdf.). GE Healthcare (2007).

12. Yagupsky P, Nolte FS. Quantitative Aspects of Septicemia. *Clin Microbiol Rev* **3**, 269-279 (1990).

13. Arabski M, Wegierek-Ciuk A, Czerwonka G, Lankoff A, Kaca W. Effects of Saponins against Clinical E. coli Strains and Eukaryotic Cell Line. *J Biomed Biotechnol*, (2012).

14. Bangham AD, Glauert AM, Horne RW, Dingle JT, Lucy JA. Action of Saponin on Biological Cell Membranes. *Nature* **196**, 952-& (1962).

15. Francis G, Kerem Z, Makkar HPS, Becker K. The biological action of saponins in animal systems: a review. *Brit J Nutr* **88**, 587-605 (2002).

16. Giudicelli S, Tomasz A. Attachment of Pneumococcal Autolysin to Wall Teichoic-Acids, an Essential Step in Enzymatic Wall Degradation. *J Bacteriol* **158**, 1188-1190 (1984).

17. Lehtonen OPJ. Inhibition of Pneumococcal Autolysis in Lysis-Centrifugation Blood Culture. *J Clin Microbiol* **24**, 493-494 (1986).

18. Daniels R*, et al.* Disulfide Bond Formation and Cysteine Exclusion in Gram-positive Bacteria. *Journal of Biological Chemistry* **285**, 3300-3309 (2010).

19. Brando B, Gohde W, Scarpati B, D'Avanzo G. The "vanishing counting bead" phenomenon: Effect on absolute CD34+cell counting in phosphate-buffered. saline-diluted leukapheresis samples. *Cytometry* **43**, 154-160 (2001).

20. Barnkob R, Kähler CJ, Rossi M. General defocusing particle tracking. *Lab Chip* **15**, 3556-3560 (2015).
